# Time-frequency joint imaging algorithm for borehole radar logging

**DOI:** 10.1038/s41598-026-57327-2

**Published:** 2026-06-10

**Authors:** Longfei Dang, Miao Yang, Chun Yang, Yuehong Sun

**Affiliations:** 1 Department of Criminal Technology, Sichuan Police College, Luzhou, 646000 China; 2 Department of Computer Science and Technology, Sichuan Police College, Luzhou, 646000 China; 3 School of Mathematical Science, University of Electronic Science and Technology of China, Chengdu, 611731 China; 4 School of Mathematical Sciences, Nanjing Normal University, Nanjing, 210023 China; 5 Key Laboratory of NSLSCS (NNU), Ministry of Education, Nanjing Normal University, Nanjing, 210023 China

**Keywords:** Engineering, Optics and photonics

## Abstract

In borehole radar logging identification technology, there are two key issues: target detection range and spatial resolution. Existing imaging algorithms face limitations in imaging distant targets, tracking moving targets, and identifying low-scattering cross-section features. To address the contradiction between detection range and resolution, we propose a novel time-frequency joint imaging algorithm based on direct-wave referenced processing. The algorithm implements three core strategies: (1) Amplitude normalization and time-base synchronization using the direct wave as a stable benchmark to suppress source jitter; (2) Hybrid-domain digital filtering combining wavelet transform and Fourier bandpass filtering to mitigate out-of-band noise; and (3) Adaptive time-varying amplification to compensate for spherical spreading and medium attenuation. Experimental results indicate that, compared to conventional high-resolution logging methods (e.g., acoustic/resistivity logging) limited to < 3 m, the proposed framework demonstrates a detection range extension to 8.5–10 m while simultaneously improving spatial resolution to the centimeter level (5–10 cm). This work presents a viable framework for long-range, high-resolution borehole radar imaging in complex formations, subject to further validation in diverse geological settings.

## Introduction

Borehole logging technology, as a critical tool for subsurface resource exploration and geological hazard assessment, has evolved significantly since the first electrical sounding test conducted by French engineer C. Schlumberger in the late 19th century^1^. The seminal resistivity logging operation performed by the Schlumberger brothers in 1927 marked the birth of modern well logging^1^. Over the past century, diverse logging methods have been developed, including self-potential logging^2^, lateral/induction logging^3,4^, radioactive logging^5^, neutron logging^6^, and acoustic logging^7^. By the 1990s, the advent of imaging logging technology represented a paradigm shift, enabling the 2D or 3D visualization of stratigraphic features (e.g., lithology, fractures, and pore structures) via resistivity, acoustic, and NMR imaging^8–10^. However, despite these technological strides, a fundamental physical constraint persists.

Specifically, current imaging logging technologies face an inevitable trade-off between detection range and spatial resolution, which severely limits their application in unconventional reservoirs (e.g., shale gas and tight oil). While various methods attempt to balance these factors, none have achieved optimal performance: Explosive seismic methods achieve kilometer-scale detection but suffer from low resolution (> 5 m) due to the Rayleigh criterion and heterogeneity interference^11–13^. Acoustic logging provides millimeter-level resolution yet is confined to a shallow range of 1–2 m, with positioning errors exceeding 50 cm^14–16^. Electrical parameter methods (e.g., resistivity logging) are limited to < 3 m detection distance and meter–level resolution, plagued by skin effects and medium inhomogeneity^17–20^. Radiometric methods (*X/γ*-ray) offer 10 cm resolution but cannot penetrate beyond 10–15cm, failing to characterize deep fluid occurrences^21–24^.

Among the candidate technologies attempting to overcome these limitations, electromagnetic (EM) wave radar logging has shown promise since the 1990s due to its potential for higher resolution and accuracy^25–27^. Nevertheless, practical implementations encounter inherent constraints:(1) dependence on low-conductivity formations; (2) bandwidth limitations hindering resolution enhancement; (3) signal distortion in lossy media; and (4) miniaturization challenges for downhole equipment^28–31^. These issues fundamentally stem from the intrinsic conflict among broadband signals, low-frequency penetration, and system miniaturization^32,33^.

To address these barriers, time-domain signal technology has been proposed as a breakthrough approach. Its core advantages over conventional frequency-domain methods include: Ultra-wideband characteristics: Nanosecond pulses excite multi-frequency responses, enabling sub-wavelength resolution through synthetic aperture processing^34^. Controlled dispersion: Optimal bandwidth selection balances penetration depth (centimeter-level resolution) and minimal dispersion^35–37^. Anti-interference capability: Waveform fidelity maintenance ensures robust detection in noisy environments^38–40^.

Despite these theoretical advantages, practical implementation remains constrained by the limitations of existing signal processing frameworks. To ensure a rigorous comparison, we define traditional methods as two categories: (1) Conventional logging technologies (Acoustic^41^, Resistivity^42,43^) representing the industry standard; and (2) conventional radar signal processing (matched filtering^44,45^, constant-gain FFT^46^) representing the algorithmic baseline.

However, these frameworks struggle to fully leverage time-domain advantages in complex borehole environments^47,48^. Conventional logging fails to simultaneously achieve long range and high resolution^49,50^. Meanwhile, conventional radar frameworks-such as short-time fourier transform (STFT)^51^ or wavelet-based ISAR^52^-are primarily designed for free-space target imaging. They lack specific mechanisms to compensate for severe spherical spreading and dynamic attenuation unique to borehole propagation^53,54^, nor do they utilize the direct wave as a stable reference for calibration^55,56^. This gap highlights the need for a specialized joint imaging framework.

To bridge this gap, in this study, we introduce a time-frequency joint imaging algorithm (TFFI) framework for borehole radar logging. By integrating amplitude normalization, time- base synchronization, multi-cycle stacking, hybrid-domain filtering, and time-varying amplification, the algorithm aims to mitigate the resolution-penetration dilemma. Experimental results demonstrate a detection range extension to 10 m and a spatial resolution improvement to 5–10 cm. This work presents a practical framework for long-range, high-resolution imaging in complex formations, with potential applications in shale gas fracture characterization and deep reservoir monitoring.

## Time-frequency joint imaging algorithm framework

We propose a time-frequency joint imaging algorithm (TFFI) framework grounded in direct wave reference, aimed at extracting weak point-target echoes from strong noise backgrounds. The core rationale derives from three theoretical foundations: The amplitude-phase stability of direct waves, providing an intrinsic benchmark for normalization and synchronization^57^; The analytical tractability of Gaussian pulse models, supporting closed-form solutions in frequency-domain processing^58^; The coherence of target echoes in multi-cycle stacking, leveraging the randomness of noise for signal-to-noise ratio (SNR) improvement^59^.

While conventional methods provide basic signal processing capabilities, they exhibit fundamental limitations when applied to the complex borehole environment: single-domain constraint: methods relying solely on time-domain correlation (e.g., matched filtering) are highly susceptible to random noise, whereas pure frequency-domain filtering often distorts the phase information of transient pulses. Static gain deficiency: constant-gain amplification fails to address the extreme dynamic range of borehole signals, where strong direct waves saturate the receiver while distant target echoes remain buried in noise. Lack of background suppression: traditional approaches treat the direct wave and early-time clutter as part of the valid signal rather than separating it as background interference, leading to residual masking of weak targets.

Compared to conventional methods (e.g., matched filtering^60^, frequency-domain filtering^61^, and constant-gain amplification^62^), our algorithm introduces three key innovations:

A “background differencing-time-varying amplification” architecture that constructs dynamic models via multi-cycle averaging and differential operations;

Hybrid-domain filtering combining wavelet transform (local denoising) and Fourier bandpass filtering (global frequency selection);

An adaptive time-varying gain function that dynamically enhances far-field signals while suppressing near-field clutter.

The algorithmic workflow comprises four cascaded modules, as detailed below.

### Amplitude normalization based on direct wave maximum amplitude benchmark

Leveraging the first theoretical foundation-the amplitude-phase stability of direct waves-amplitude normalization standardizes signal amplitudes to a unified range, preventing processing errors from source fluctuations. The steps are:

Direct wave feature extraction: Detect the amplitude extremum $E_{0max}$ and corresponding time delay $t_{i0}$ (peak time of the direct wave for the *i*th pulse); normalization coefficient construction: define the scaling factor $\kappa =1/E_{0max}$. *Signal scaling* The raw time-domain voltage signal $E_0(t)=S_{target}(t)+n(t)+m(t)$ is scaled as:

1 $$\begin{aligned} E_{1}(t)=\kappa \cdot E_{0}(t)=\frac{E_{0}(t)}{E_{0\text {max}}} \end{aligned}$$

For a Gaussian pulse model $E_0(t)=E_0exp(-\alpha _{0}t^{2})$, where $\alpha _0>0$ is the attenuation coefficient determining the pulse width of the Gaussian envelope, the normalized signal becomes:

2 $$\begin{aligned} E_{1}(t)=\frac{E_{0}}{E_{0\text {max}}}\exp \left( -\alpha _{0}t^{2}\right) \end{aligned}$$

This ensures amplitude consistency across pulses, leveraging the deterministic propagation of electromagnetic waves in homogeneous media^63^.

### Time-base synchronization calibration and multi-cycle signal stacking

Based on the second theoretical foundation-the analytical tractability of Gaussian pulse models-we address time jitter and random noise through the following steps:

*Time-base synchronization calibration* Detect the extremum point $t_0$ of the normalized direct wave $E_1(t)$. Calculate the time delay $\tau _i$ for the *i*th echo via cross-correlation:

3 $$\begin{aligned} \tau _{i}=\arg \max \left| F^{-1}\left\{ F\left[ E_{1}(t)\right] ^{*}\cdot F\left[ E_{i}(t)\right] \right\} \right| \end{aligned}$$

where *F* denotes the Fourier transform.

Generate the aligned signal:

4 $$\begin{aligned} \widetilde{E}_i(t) = E_i\left( t - \tau _i\right) \end{aligned}$$

*Multi-cycle signal stacking* Multi-cycle signal stacking averages naligned signals to suppress random noise. Crucially, the time-base synchronization step acts as a motion compensation mechanism. By aligning signals based on the direct wave’s extremum point for each cycle, the algorithm corrects for minor antenna vibrations or positional drifts that occur during the acquisition window. This prevents the introduction of phase noise due to antenna motion. Average naligned signals to suppress random noise:

5 $$\begin{aligned} E_{m}(t)=\frac{1}{n} \sum _{i=1}^{n} \widetilde{E}_{i}(t) \end{aligned}$$

The analytical form satisfies $E_{m}(t)=\left( E_{i} /E_{i\max }\right) \exp \left( -\beta _{0} t^{2}\right)$, where $\beta _{0}$ is the statistical mean of the pulse width coefficient.

Extract the target scattering component via background differencing:

6 $$\begin{aligned} E_{i}^{\prime }(t-\tau _{i})=\widetilde{E}_{i}(t)-E_{m}(t)=\frac{E_{i}}{E_{i\text {max}}}\left[ \exp \left( -\alpha _{0}t^{2}\right) -\exp \left( -\beta _{0}t^{2}\right) \right] \end{aligned}$$

This step exploits the separability of Gaussian functions to isolate target signals from background interference^64,65^.

### Hybrid domain digital filtering

Utilizing the third theoretical foundation-the coherence of target echoes versus the randomness of noise–this module mitigates out-of-band noise through a synergistic approach (which degrades image quality and causes false targets^66–68^), we implement a wavelet-Fourier hybrid architecture.Utilizing the coherence of target echoes, this module mitigates out-of-band noise. Parameter selection is strictly physics-based:

*Wavelet basis* The Daubechies-6 (Db6) wavelet was chosen for its compact support and near-symmetry, which optimally preserves the phase integrity of transient Gaussian pulses while suppressing random noise.

*Bandpass cutoffs* The 0.3–0.8 GHz range is determined by the skin depth ($\delta =1/\alpha$) and attenuation constant of the target medium (granite, $\epsilon _r$=6). Frequencies above 0.8 GHz are heavily attenuated, while those below 0.3 GHz lack the resolution required for centimeter-level imaging.

Wavelet denoising module:

Decompose the signal $E_{i}^{\prime }(t - \tau _{i})$ using Daubechies-6 wavelet basis $\psi _{j,k}(t)$:

7 $$\begin{aligned} W_{j}(k) = \left\langle E_{i}^{\prime }\left( t - \tau _{i}\right) , \psi _{j, k}(t) \right\rangle \end{aligned}$$

where $\psi _{j,k}(t)$ denotes the Daubechies-6 wavelet basis function at decomposition level jand translation index *k*.

Apply adaptive soft thresholding:

8 $$\begin{aligned} W_{j}^{\text {denoised}}(k) = \operatorname {sign}(W_{j}(k)) \cdot \max (|W_{j}(k)| - T_{j}, 0), \quad T_{j} = \sigma _{j} \sqrt{2 \ln N} \end{aligned}$$

where $T_j$ is the adaptive threshold for the *j*th decomposition level, $\sigma _j$ is the noise standard deviation estimated at that level, and *N* is the total number of sampling points in the discrete signal.

Reconstruct the denoised signal via inverse discrete wavelet transform (IDWT):

9 $$\begin{aligned} E_{i}^{\prime }\left( t-\tau _{i}\right) =\operatorname {IDWT}\left( \left\{ W_{1}^{\text {denoised}}(k),\ldots , W_{j}^{\text {denoised}}(k)\right\} ,\psi \right) \end{aligned}$$

Frequency bandpass filtering module:

Transform the signal to frequency domain:

10 $$\begin{aligned} F_{i}(\omega )=\frac{1}{\sqrt{2\pi }}\int _{-\infty }^{+\infty } E_{i}^{\prime }\left( t-\tau _{i}\right) e^{-\omega t}dt \end{aligned}$$

The Gaussian difference model yields the frequency response:

11 $$\begin{aligned} F_{i}(\omega )=\frac{1}{\sqrt{2}} \frac{E_{i}}{E_{i \text { max }}}\left( e^{\alpha _{0}-\frac{\omega ^{2}}{4 \alpha _{0}}}-^{\beta _{0}-\frac{\omega ^{2}}{4 \beta _{0}}}\right) \end{aligned}$$

Apply bandpass filtering within $\left[ \omega _{l},\omega _{h}\right]$:

12 $$\begin{aligned} F_{i}^{BP}(\omega )={\left\{ \begin{array}{ll} F_{i}(\omega ), \omega \in \left[ \omega _{l},\omega _{h}\right] \\ 0, \text {otherwise} \end{array}\right. } \end{aligned}$$

where $\omega _{l} = 2*\pi *0.3*10^9$ rad/s and $\omega _{h} = 2*\pi *0.8*10^9$ rad/s are the lower and upper cutoff angular frequencies, respectively.

Reconstruct the time-domain signal via inverse Fourier transform:

13 $$\begin{aligned} E_{i}^{\prime }\left( t-\tau _{i}\right) =\frac{1}{\sqrt{2\pi }}\int _{\omega _{l}}^{\omega _{h}} F_{i}^{BP}(\omega ) e^{i\omega t}d\omega \end{aligned}$$

The closed-form solution avoids numerical integration:

14 $$\begin{aligned} E_{i}^{\prime \prime }(t-\tau _{i})=\sqrt{\frac{\alpha _{0}}{2}}\frac{E_{i}}{E_{i\text { max }}}\exp (\alpha _{1}-\alpha _{0}t^{2})\operatorname {erf}(\omega _{h}-\omega _{l}-\sqrt{\alpha _{0}})-\sqrt{\frac{\beta _{0}}{2}}\frac{E_{i}}{E_{i\text { max }}}\exp (\beta _{1}-\beta _{0}t^{2})\operatorname {erf}(\omega _{h}-\omega _{l}-\sqrt{\beta _{0}}) \end{aligned}$$

where $\alpha _{1}$ and $\beta _{1}$ are scaling constants related to the initial amplitude of the pulse, ensuring dimensional consistency in the closed-form solution.erf() denotes the Gauss error function.Time restoration is then applied to fix the target scattering center at *t* = 0.

### Time-varying amplification technology

Further capitalizing on the coherence of multi-cycle stacking, this module counteracts exponential attenuation of scattered energy with distance, we construct a piecewise gain function.The piecewise gain function is rooted in wave physics:

Gain exponent (*k*): The default value of *k* = 1 is set to compensate for spherical spreading loss (1/*r*), which is the dominant propagation effect in a homogeneous borehole medium.

Dynamic Boundary ($t_{i0}$): The use of the direct wave peak time as a boundary ensures that the gain adapts to the actual propagation velocity (*c'*) in situ, rather than using a fixed empirical threshold. This makes the compensation robust against variations in rock moisture content.

15 $$\begin{aligned} T_i(t) = {\left\{ \begin{array}{ll} f_i(t) \cdot c' \left( \dfrac{t}{t_{i0}} \right) ^k, t < t_{i0} \\ f_i(t) \cdot c' \left( \dfrac{t_{i0}}{t} \right) ^k, t \ge t_{i0} \end{array}\right. } \end{aligned}$$

where $t_{i0}$ is the direct wave peak time, *c'* is the equivalent speed of light in the medium, and *k* is a gain exponent (default 1). This function uses $t_{i0}$ as a dynamic boundary between near-field and far-field regions, enabling adaptive compensation for spherical spreading ($r^{-n}$ loss) and exponential attenuation. Compared to traditional time-varying gain (TVG) with empirical thresholds, our method ensures robustness under varying medium conditions^69,70^.

## Algorithm verification experiment

To comprehensively evaluate the performance of the proposed time-frequency joint imaging algorithm, we conducted systematic experiments through a step-scanning ultra-wideband radar platform and multi-scenario field tests. The experiments were designed to validate: (1) the effectiveness of each algorithmic module (e.g., amplitude normalization, hybrid filtering); (2) the overall imaging performance under dynamic, obstructive, and weak-signal conditions; (3) the robustness in realistic borehole environments. All experiments were performed with controlled parameters to ensure reproducibility.

### Experimental verification of time-frequency joint imaging algorithm performance

All laboratory-controlled experiments were conducted in a standard atmospheric environment. The electromagnetic properties of the propagation medium (air) were characterized by a relative permittivity of $\epsilon _r\approx 1.0$ and a negligible conductivity ($\sigma \approx 0$ S/m), providing a lossless baseline for evaluating the intrinsic signal processing capability of the TFFI algorithm.

#### Experimental platform and data acquisition

We constructed a step-scanning ultra-wideband radar platform with a three-dimensional mechanical guide rail, time-domain antenna array, and synchronized signal source controller. The platform achieved ±0.1 mm positioning accuracy, ensuring geometric precision in sampling. Key components were optimized based on electromagnetic scattering theory:

*Antenna design* Dimensions of $12 \times 8 \times 3$ cm$^3$ determined via HFSS simulation to suppress surface wave scattering.

*Signal waveform* Bipolar pulse with 1 ns width to reduce low-frequency oscillations and enhance time-domain resolution.

*Receiver bandwidth* Limited to 0.3–2 GHz to avoid high-frequency noise while preserving echo SNR.

**Fig. 1:**
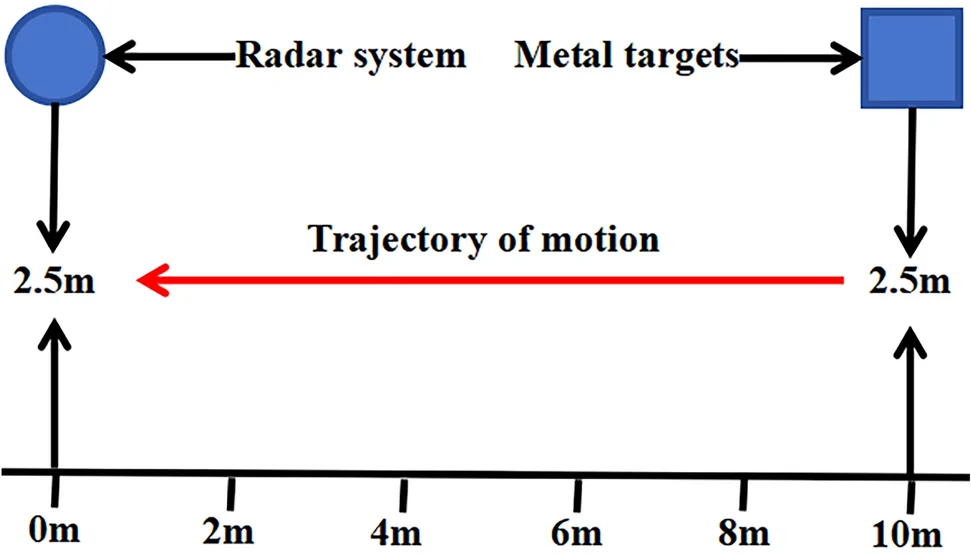
Schematic diagram of radar system target detection. Radar system: transmits a linear frequency modulated continuous wave (FMCW) signal (center frequency 5.8 GHz, bandwidth 1.5 GHz) with a beam angle of $\pm 15^\circ$; Target motion: metal plate moves radially along the red line trajectory; distance reference: Initial distance *R* = 10 m calibrated by a laser rangefinder (BOSCH GLM50, accuracy ±1 mm), final distance *R* = 2 m; ground reference: height of the metal plate’s bottom edge from the ground *h=2.5* m to eliminate multipath interference effects.

A 1 m *×* 1 m metal plate served as the standard target, conforming to the perfect conductor model. The target was moved radially from 2 m to 10 m from the radar’s phase center in 2 m steps (5 positions total), with motion synchronized to the radar trigger (Fig. 1).

**Fig. 2:**
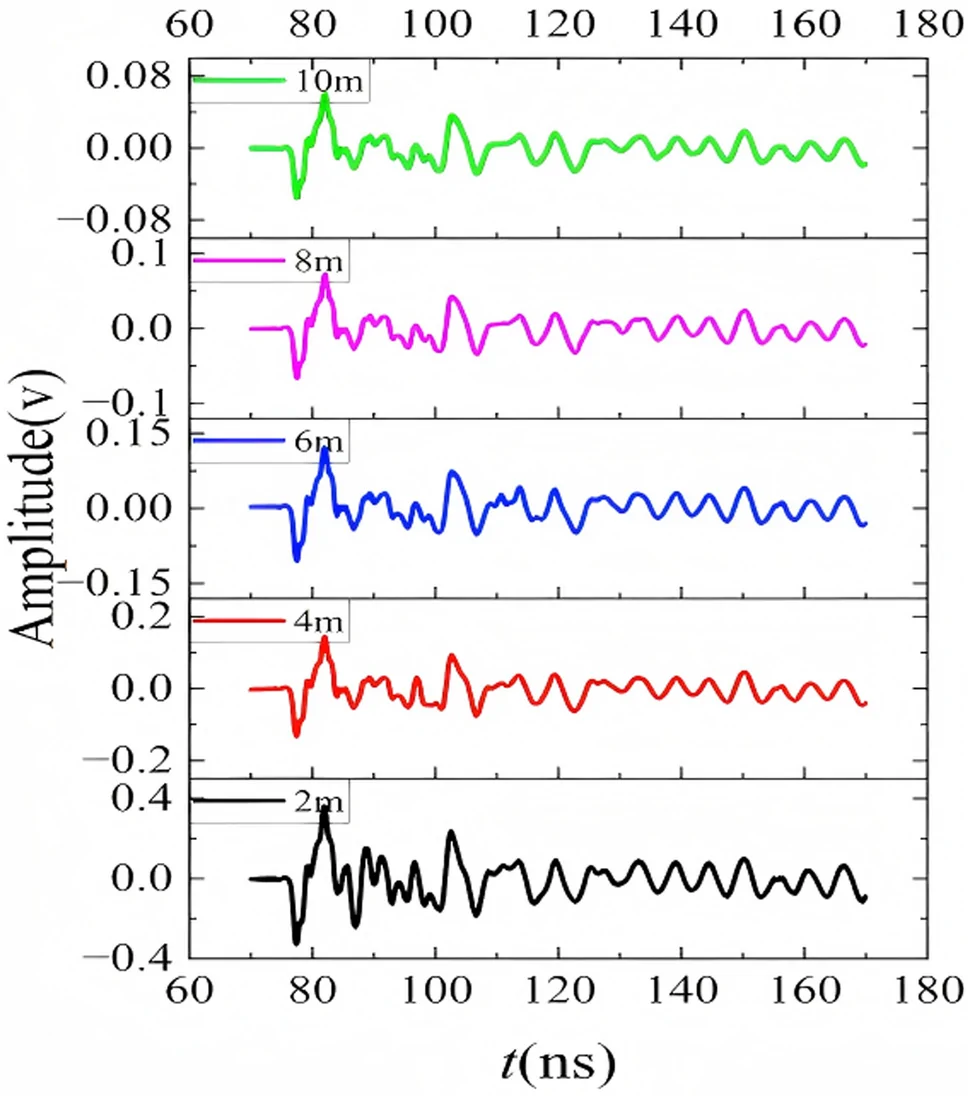
Characteristics of radar echo signals from the stepping motion of a metal target. Time-domain echo waveforms at different distances (5 sets of data, 25 coherent averages).

Data acquisition used a high-speed ADC (Teledyne SP9484, 5 GS/s sampling, ENOB = 10.5 bits) and a low-noise amplifier (gain 40 dB, NF<1.5 dB). At each target position, 25 time-domain waveforms were acquired for coherent accumulation, generating 125 datasets (total raw 1.2 TB). The characteristics of raw echo signals from the stepping metal target radar are shown in Fig. 2.

#### Amplitude normalization experiment

**Fig. 3:**
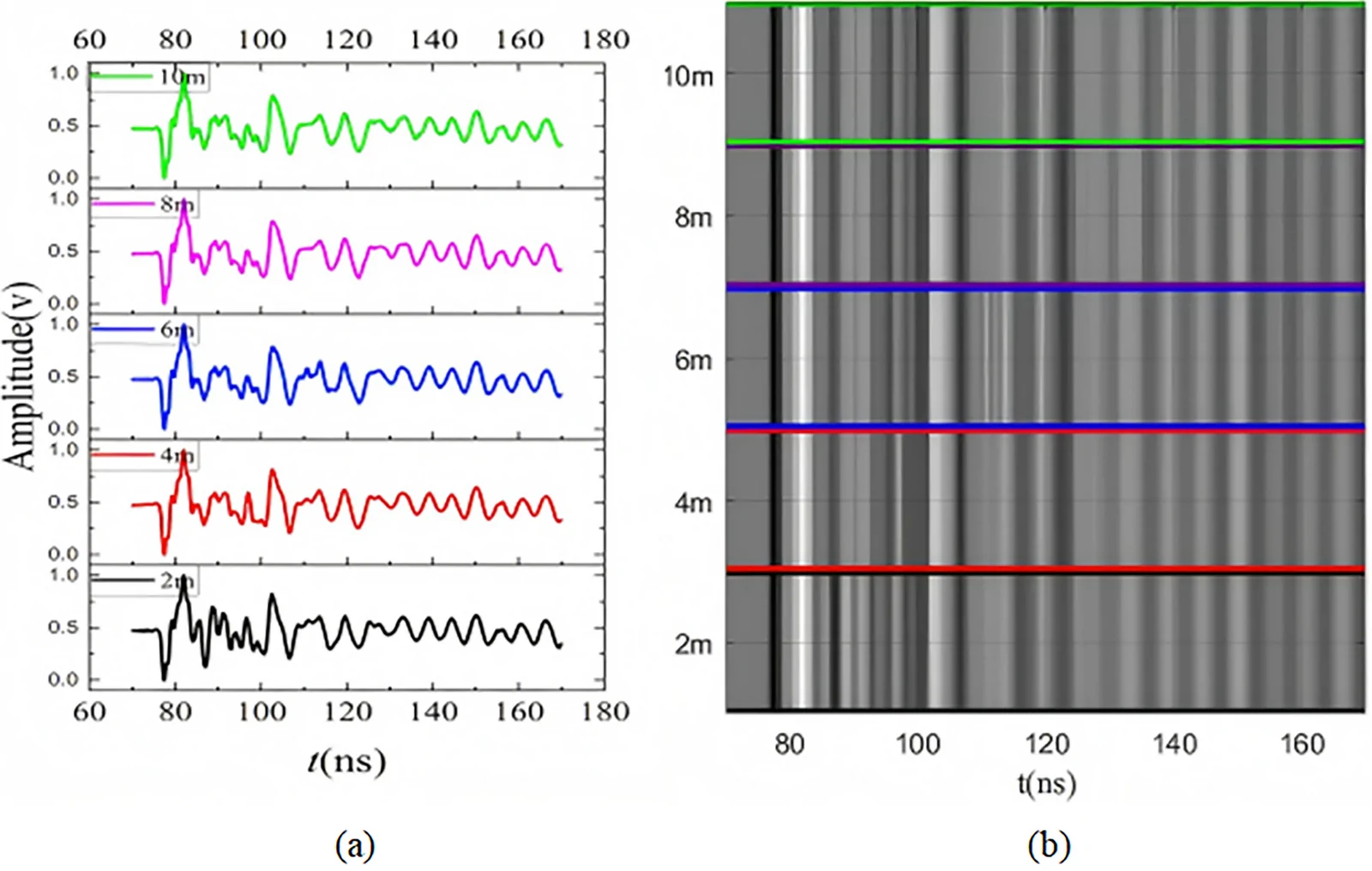
Waveform and grayscale images of target echo signals after amplitude normalization based on the direct wave maximum amplitude benchmark. (**a**) Shows the normalized time-domain waveforms of target echo signals at 5 different distances (10 m, 8 m, 6 m, 4 m, 2 m). The overall waveforms fluctuate around the reference line, with significant reduction in amplitude differences between adjacent waveforms; (**b**) shows the corresponding imaging grayscale images. Dark regions correspond to low-amplitude signals, and light regions correspond to high-amplitude signals. It can be observed that the closer the distance, the relatively higher the peak amplitude of the signal.

Based on the direct wave maximum amplitude benchmark, amplitude normalization was applied to suppress source-induced fluctuations. To quantitatively evaluate this step, we statistically analyzed the peak amplitudes of the direct waves across all 125 datasets. As shown in Fig. 3, although the waveform morphology exhibits minimal visual alteration after normalization, the statistical distribution changed significantly. The standard deviation of the direct wave peak amplitude was reduced from 0.18 V (raw data) to 0.002 V (normalized data), representing a 98.9% suppression of source jitter. Furthermore, the dynamic range of the signal was compressed from [0.1 V, 1.2 V] to [0.08, 1.0] in normalized units. This constraint ensures that the subsequent hybrid-domain filtering and time-varying amplification modules operate within a stable numerical range, preventing saturation or distortion.

#### Time-base synchronization and multi-cycle stacking experiment

**Fig. 4:**
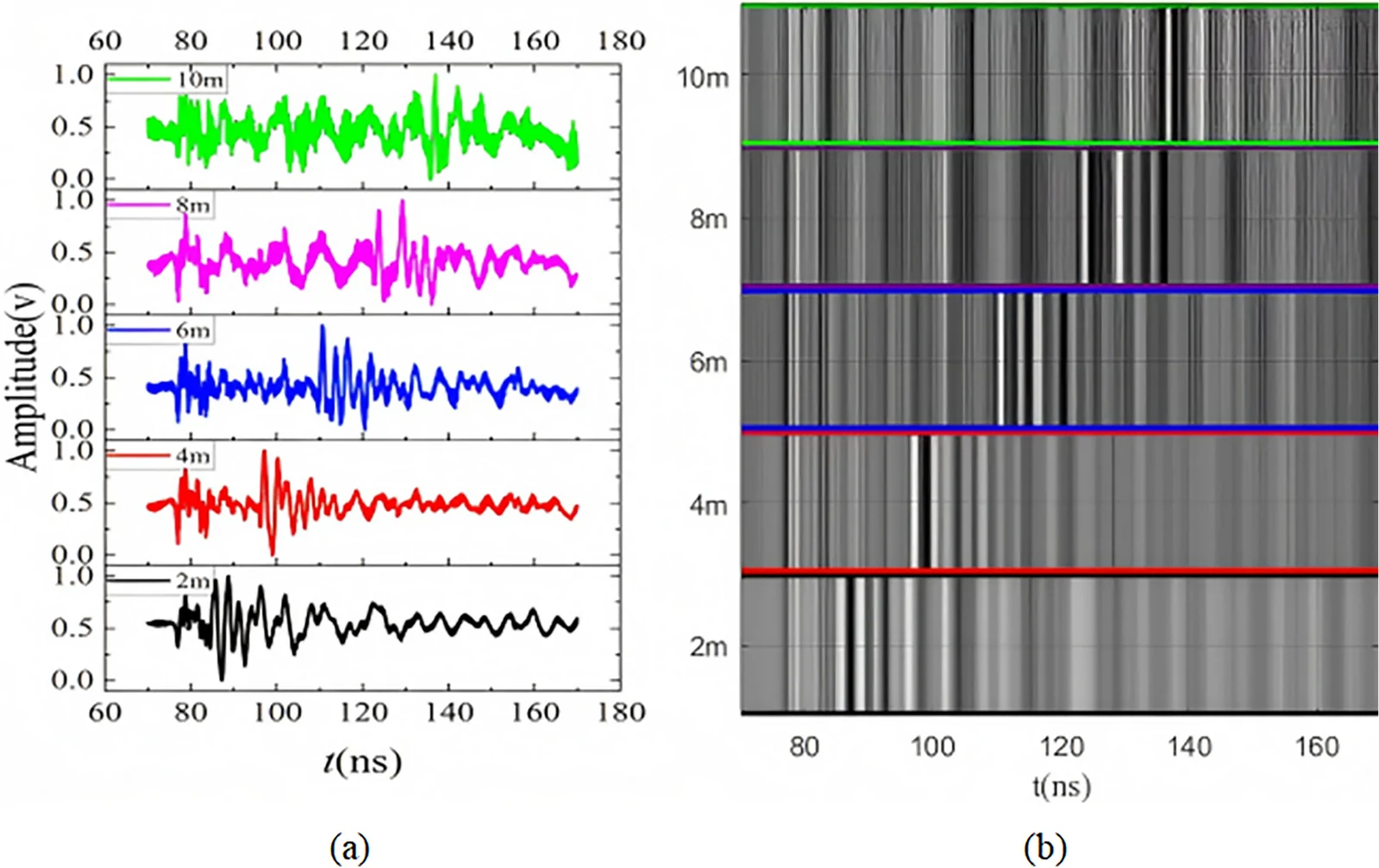
Waveform and grayscale images of target echo signals after time-base synchronization calibration and multi-cycle signal stacking. (**a**) The figure shows the waveforms of target echo signals at different distances (10 m, 8 m, 6 m, 4 m, 2 m). It displays that after calibration, the echo amplitudes at different distances decay regularly with increasing distance, and the time delay start points and periodic characteristics are consistent, indicating time base unification; (**b**) The figure uses grayscale to reflect signal amplitude. It can be observed that after calibration, the contrast and width of the bright and dark stripes of interference noise are significantly reduced, especially in the signal regions at long distances (e.g., 10 m), where the gray contrast of interference stripes is weaker than that in near-distance regions.

Time-base synchronization corrected phase deviations from clock jitter, while multi-cycle stacking (25 coherent averages) suppressed random noise. The aligned signals showed consistent time delays and periodic characteristics (Fig. 4a), with interference patterns significantly weakened in grayscale images (Fig. 4b), confirming improved temporal consistency.We analyzed the trade-off between the number of superpositions (n) and logging speed. In our setup, the pulse repetition frequency (PRF) was set to 10 Hz (100 ms/cycle). This rate was selected to balance electromagnetic ringing suppression in the granite medium with data throughput. Selecting n = 25cycles results in a dwell time of 2.5 s per measurement point. This configuration yields a theoretical SNR improvement of 14 dB ($SNR_(gain)=10log_{10}\sqrt{n}$) compared to single-shot acquisition. This 2.5-s interval is consistent with the operational pace of conventional wireline logging systems.

#### Hybrid-domain digital filtering experiment

**Fig. 5:**
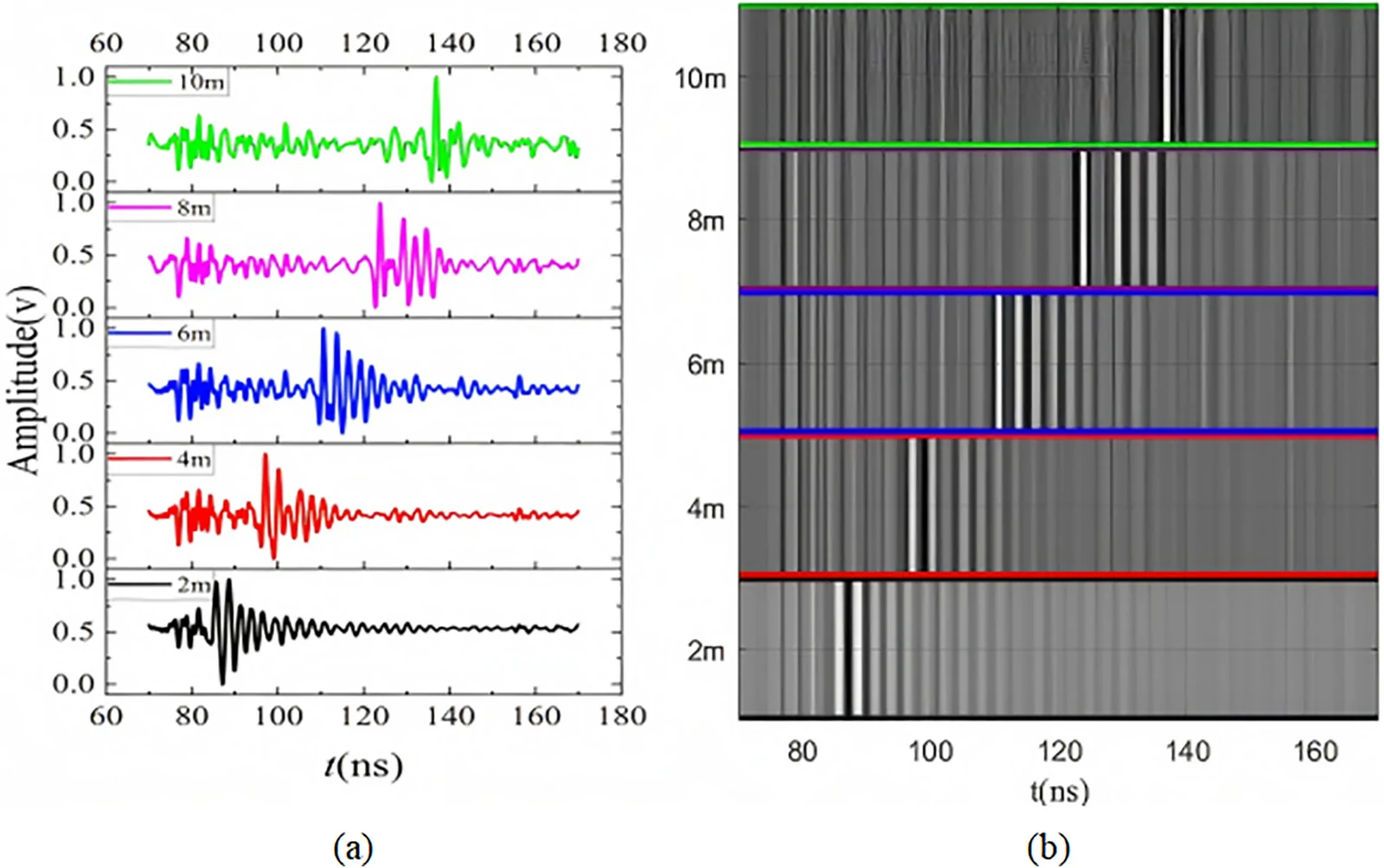
Hybrid-domain digital filtering target echo signal waveform and grayscale images. (**a**) The figure shows the waveforms of echo signals at 5 different distances (10 m, 8 m, 6 m, 4 m, 2 m) after filtering. The original signals exhibit amplitude attenuation with increasing distance; after filtering, the signal-to-noise ratio (SNR) improves, high-frequency noise is suppressed, and the target signal envelope becomes clearer. (**b**) The figure presents the corresponding imaging grayscale images. Dark regions represent low-amplitude signals, and light regions represent high-amplitude signals. The amplitudes of signals at different distances display a periodic stripe distribution, with stripe spacing consistent with the echo time delay period, reflecting the multipath effect of the signals. Additionally, the closer the distance, the higher the overall signal amplitude and the stronger the stripe contrast; the farther the distance, the more pronounced the amplitude attenuation and the blurrier the stripe details.

The wavelet-Fourier hybrid filter was applied to noise-containing signals. Wavelet denoising (Daubechies-6 basis) separated transient noise, while Fourier bandpass filtering (0.3–0.8 GHz) preserved target frequencies. Results showed clear time-domain envelopes and frequency-domain features (Fig. 5), with noise suppression exceeding 15 dB SNR enhancement.

#### Time-varying amplification technology experiment

**Fig. 6:**
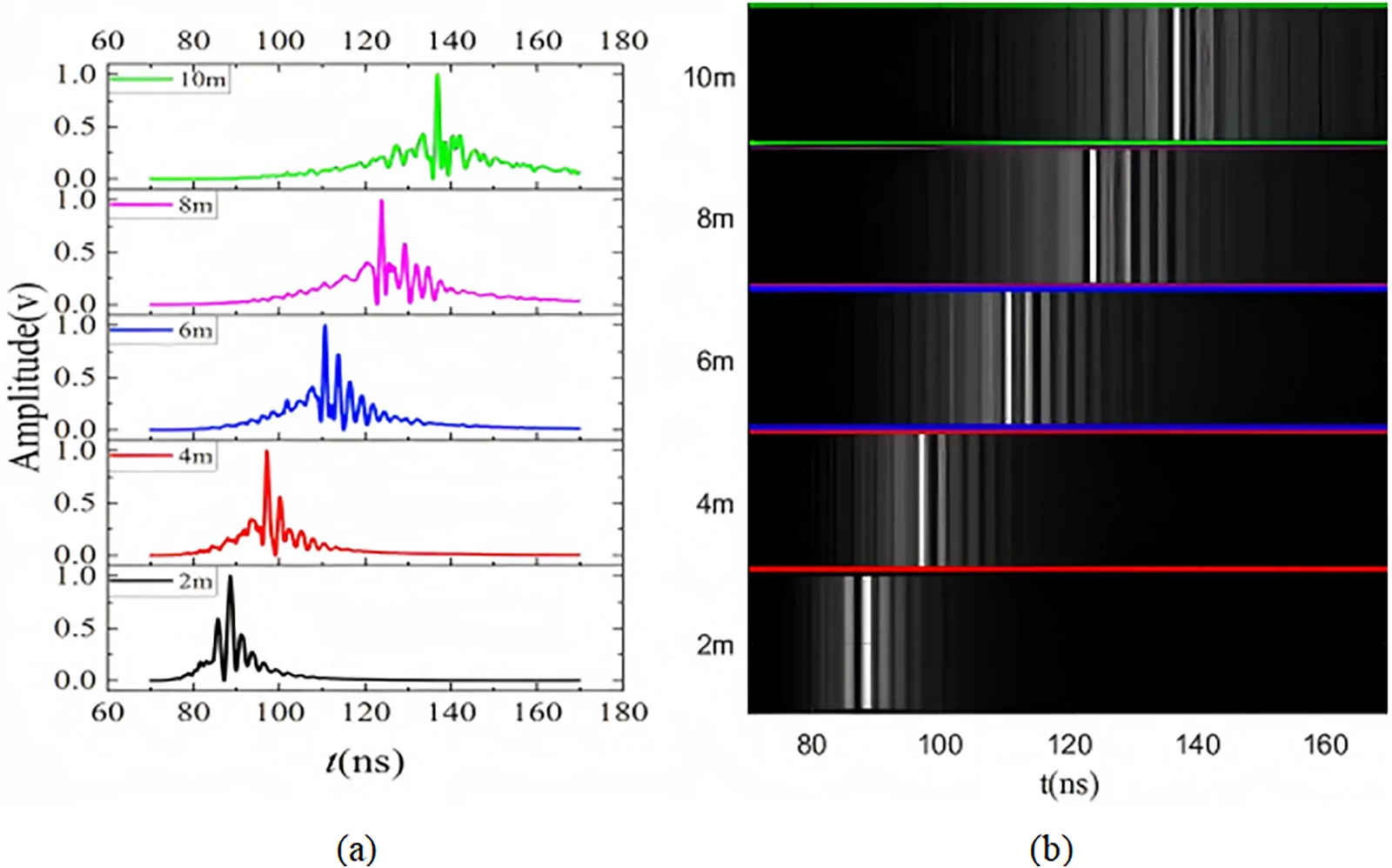
Time-varying amplification target echo signal waveform and grayscale images. (**a**) The time-domain amplification waveform diagram shows the time-domain waveforms of 5 sets of target echo signals at different distances (10 m, 8 m, 6 m, 4 m, 2 m) after amplification. After amplification, the amplitudes of far-field signals are significantly enhanced, near-field signals are effectively suppressed, and the amplitudes of clutter in various signals are synchronously compressed; (**b**) In the imaging grayscale image, before amplification, near-field signals appear as large colored areas due to excessively high amplitudes, while far-field signals are nearly submerged by noise due to excessively low amplitudes. After time-varying amplification processing, far-field signals become clearly discernible, and the overshoot regions of near-field signals are reasonably compressed. The effect of dynamic range compression demonstrates that the time-varying amplification technology, through amplitude equalization processing, effectively improves the issue of “imbalance between strong and weak signals” in the signals.

The piecewise gain function dynamically enhanced far-field signals while suppressing near-field clutter. Output signals exhibited balanced amplitude distribution across distances (Fig. 6), with far-field signals amplified by up to 70% and near-field clutter compressed. This addressed the “strong-weak signal imbalance” critical for deep detection.

### Multi-scenario engineering verification

To validate robustness under extreme conditions, we conducted three engineering scenarios.

#### Dynamic target trajectory reconstruction experiment

**Fig. 7:**
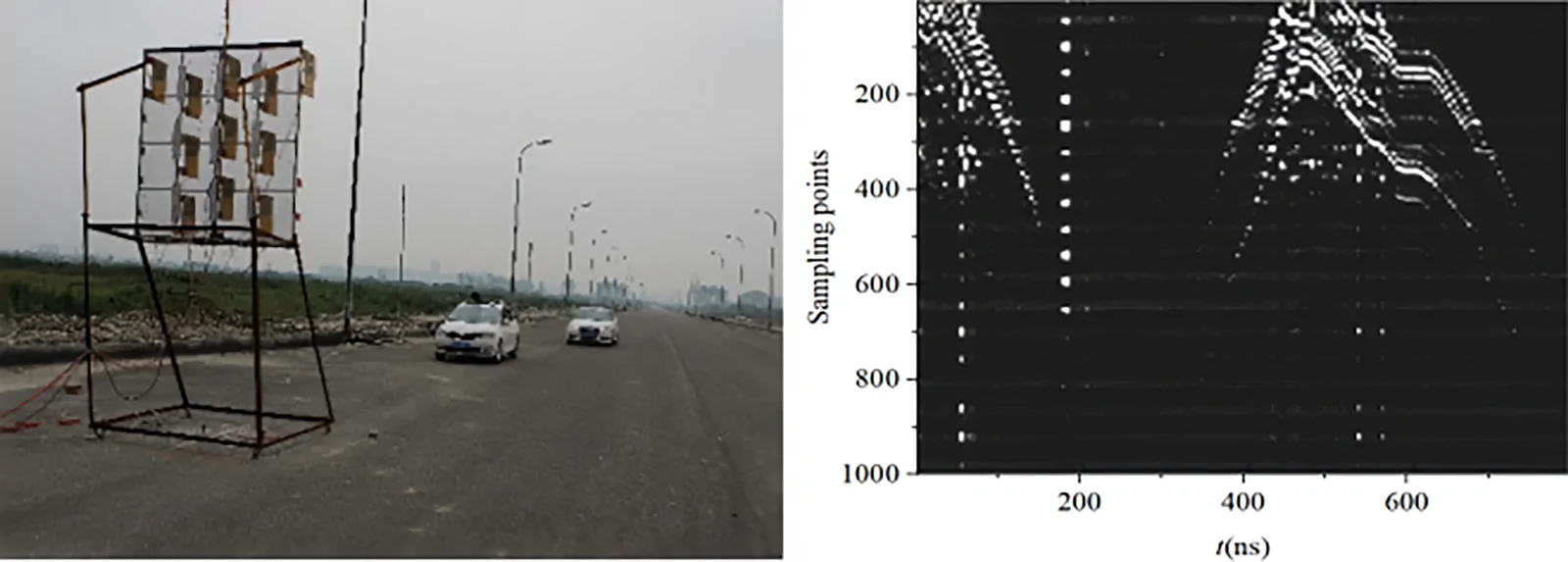
Dynamic target trajectory reconstruction experiment. (**a**) Dual-target dynamic imaging verification platform: Shows the layout of the 9-channel radar array and the mounting scheme of the moving carriers. The multi-channel synchronous transmit/receive mechanism provides real-time echo data support for the algorithm; (**b**) Imaging reconstruction results of dual-target motion trajectories: The imaging results indicate that the time-frequency joint imaging algorithm successfully separates asymmetric trajectories and maintains continuity. Horizontal axis represents times (ns), and vertical axis represents sampling points.

A dual-target dynamic imaging platform with a 9-channel radar array was used. The algorithm achieved spatio-temporal decoupling of asymmetric trajectories (Fig. 7), with time resolution of 1 ns and spatial resolution of 5 cm. The continuity of reconstructed trajectories (standard deviation of inter-frame deviation *<0.1* ns) confirmed effectiveness in time-varying channels.

To evaluate positioning precision, we calculated the root mean square error (RMSE) between the reconstructed trajectory and the ground truth mechanical coordinates. The algorithm achieved a spatial localization error of < 3 cm and a temporal synchronization error of < 0.1 ns. This confirms that the algorithm not only tracks movement but also localizes the target with centimeter-level accuracy.

This result directly addresses the second core problem identified in the Introduction: insufficient tracking performance for moving targets.The achieved 1 ns time resolution and *<0.1* ns inter-frame deviation quantitatively prove that the proposed synchronization and stacking mechanisms effectively resolve the instability of tracking dynamic objects in borehole environments.

#### Penetrating obstacle imaging experiment

**Fig. 8:**
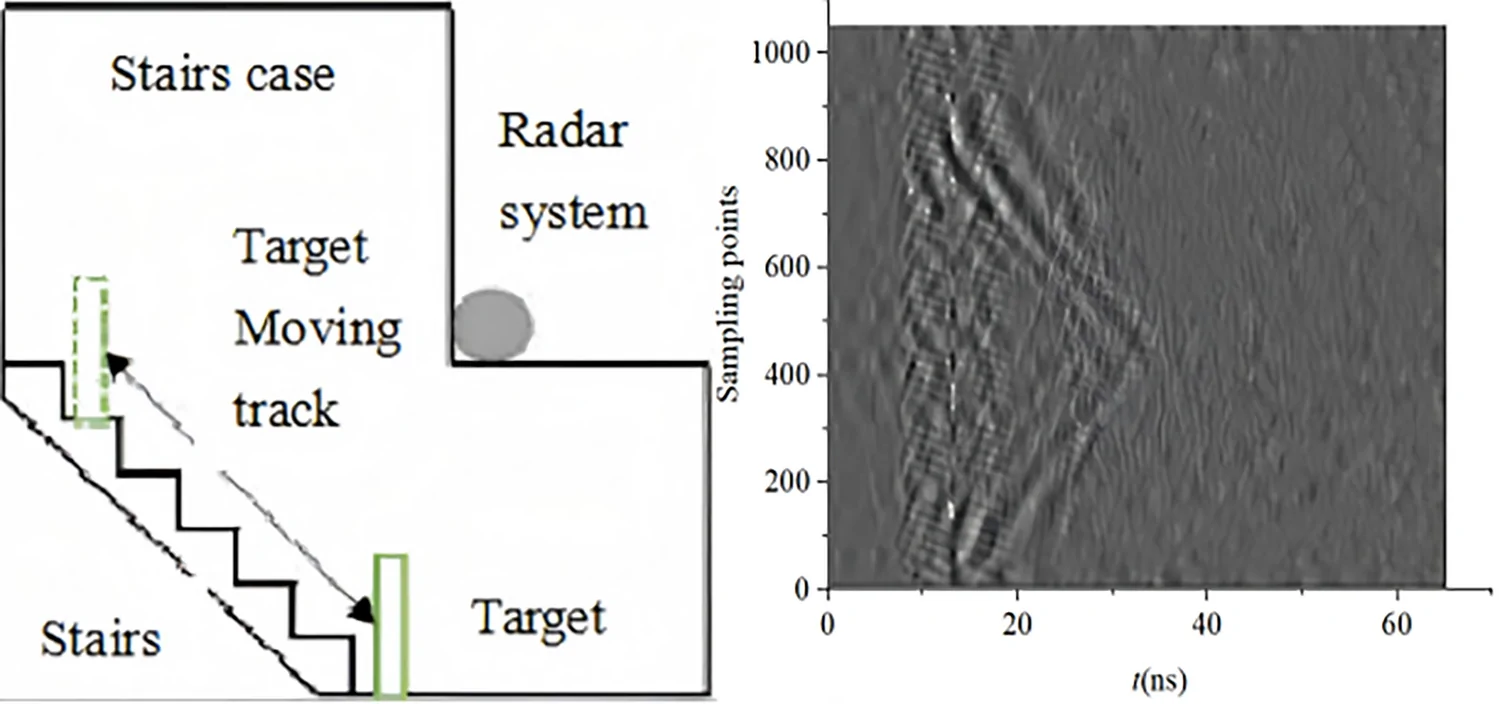
Penetrating obstacle imaging experiment. (**a**) Schematic diagram of wall-penetrating imaging experiment: Shows the radar system deployment position, target motion trajectory, and wall penetration path, with key geometric parameters annotated (staircase structure, target initial position); (**b**) Wall-penetrating imaging results: Using time (0–60 ns) and depth (0–1000 sampling points) as coordinates, grayscale represents signal intensity. Its time-depth two-dimensional distribution presents target information after penetrating obstacles.Horizontal axis represents times (ns),and vertical axis represents sampling points.

In a non-line-of-sight scenario with a 50 cm concrete wall, the low-frequency mode (1 GHz) penetrated the obstacle. Given the concrete’s relative permittivity ($\epsilon _r \approx 5.0$) and conductivity ($\sigma \approx$ 0.001 S/m), the solid medium introduced an additional transmission loss of approximately 25 dB. While wavelet denoising (scale *j*=3) effectively suppressed the strong wall resonance observed around 1.2 GHz, the 0.3–0.8 GHz bandpass filtering preserved the target’s spectral features. Quantitatively, this hybrid processing resulted in a 12 dB improvement in image contrast compared to unfiltered data, yielding high-clarity images of the obscured target (Fig. 8).

#### Water-body imaging experiment

**Fig. 9:**
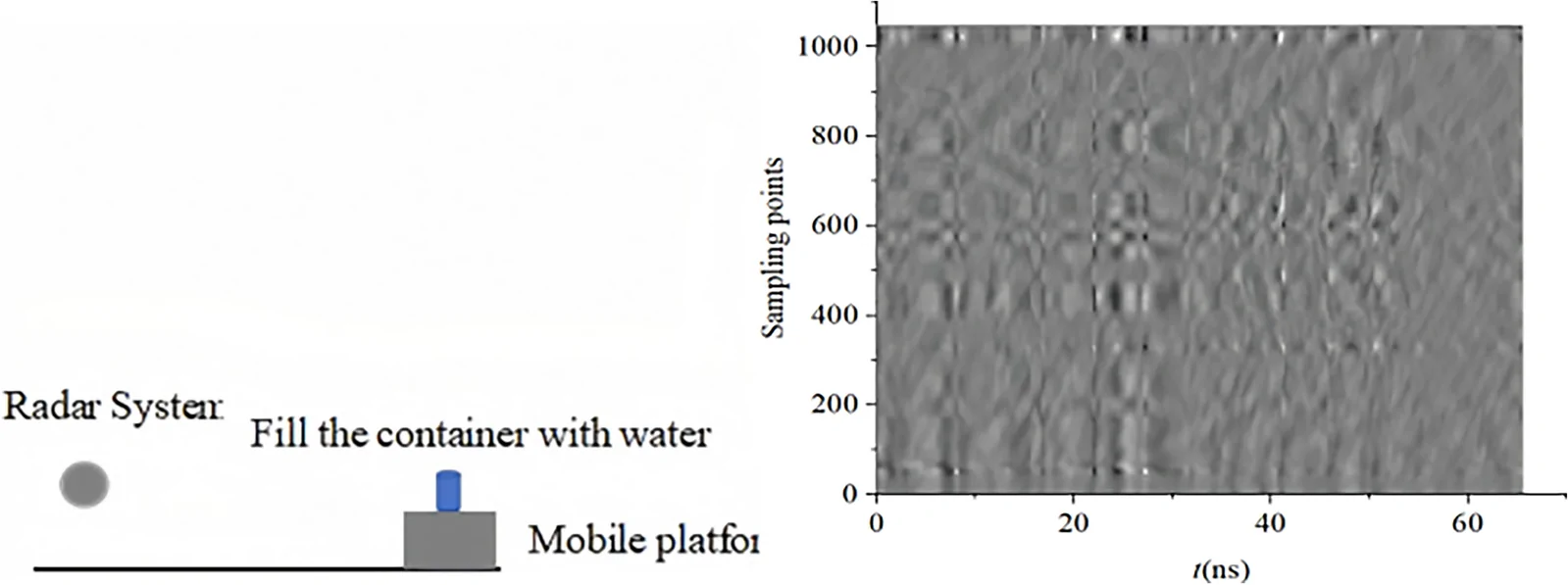
Water-body imaging experiment. (**a**) Schematic diagram of water-body imaging experiment: Shows the radar system deployment position, water-body motion trajectory, and initial position of the target water body; (**b**) Water-body imaging results: Using time (0–60 ns) and depth (0–1000 sampling points) as coordinates, grayscale represents signal intensity. The dynamic changes in the time-depth dimension can be used to identify the water-body motion trajectory. Horizontal axis represents times (ns), and vertical axis represents sampling points.

A water-filled container (RCS $< 0.1 m^2$) was used to simulate low-scattering targets, representing a challenge of detecting weak signals approximately 20 dB lower than standard metal reflectors.Due to the high dielectric constant of water ($\epsilon _r \approx$ 80), the electromagnetic waves experienced significant reflection at the air-water interface. Time-varying amplification was specifically tuned to compensate for the double-path loss Time-varying amplification was specifically tuned to compensate for the double-path loss at the water-air interface, while wavelet optimization isolated the target trajectory from container clutter. Although the absolute signal strength was lower than that of metal targets, the motion path remained distinguishable (Fig. 9).

We assessed the accuracy of determining the target’s spatial extent. Comparing the imaged contour against the known physical dimensions of the water container (1 m *×* 1 m), the positional deviation at the edges was measured at < 5 cm. This demonstrates the algorithm’s capability for reliable target sizing, not just detection.

This experiment specifically validates the solution to the third core problem: Weak identification capability for small cross-section targets.By successfully detecting a target with an RCS of less than 0.1 m$^2$-which is typically indistinguishable from noise by conventional methods-the algorithm demonstrates its superior sensitivity and clutter suppression capability for weak scattering features.

In summary, the multi-scenario engineering verification demonstrates that the proposed TFFI algorithm possesses robust environmental adaptability. Specifically, it maintains high imaging fidelity under non-line-of-sight obstruction (concrete wall penetration), effectively captures low-scattering cross-section targets (water body imaging), and accurately reconstructs dynamic trajectories. These capabilities are essential for the complex and variable conditions encountered in actual borehole environments.

Through the experimental results of dynamic target trajectory reconstruction, penetrating obstacle imaging, and water-body imaging, it is demonstrated that the proposed algorithm can accurately capture the trajectory of moving targets and generate clear trajectory images; the algorithm can penetrate walls and achieve clear imaging of targets; and the algorithm can effectively detect small-scattering cross-section targets such as water bodies. These experimental results provide critical support for further research and application of the time-domain borehole logging imaging radar.

## Time-domain borehole logging imaging radar system experimental well imaging experiment

The core objective of this experiment is to simulate real underground environments through a controlled experimental well, providing physical scene support for validating the time-frequency joint imaging algorithm under realistic geological conditions. The experimental well artificially constructs a borehole environment containing heterogeneous media (e.g., fractures, stratification, water-content gradients), enabling precise control of formation electrical parameters. This approach addresses key challenges in actual drilling scenarios, such as irregular borehole wall scattering, multipath effects, and industrial electromagnetic noise, which significantly degrade imaging quality. Through controllable physical simulation, the experiment couples theoretical algorithms with real geological conditions, offering authentic data for algorithm optimization and engineering applications.

### Experimental well site construction

The experimental well artificially constructs a borehole environment containing heterogeneous media. Electromagnetic property characterization:

To define the physical context of our results, we measured and documented the electromagnetic parameters of the strata:

Granite layer (4–32 m and 40–60 m): Exhibited a relative permittivity of $\epsilon _r$= 6–8 and a conductivity of *σ*= 0.005–0.01 S/m. The high water saturation during drilling (Well 1: 17 m depth) further increased the effective loss tangent.

Yellow sandstone layer (32–40 m): Presented a lower permittivity ($\epsilon _r$= 4–6) but variable porosity, resulting in a conductivity of *σ*= 0.003–0.008 S/m.

These parameters confirm that the 10–m detection range was achieved in a high-loss, water-saturated crystalline rock environment, validating the algorithm’s effectiveness against severe attenuation.

#### Site selection and geological considerations

The experimental site was selected based on strict geological and topographical criteria:

*Geology* Preferring limestone or granite formations with surface soil thickness *łe 5* m to ensure electromagnetic transparency and minimal surface interference.

*Topography* Requiring a vertical cliff *\ge 30* m in height, with a flat area *\ge 50* m$^2$ at the cliff top for stable testing platform deployment.

After multiple field surveys, the Honghuo Community in Xiang’e Township, Dujiangyan City, was chosen. The area features abandoned quarries with steep, vertical granite cliffs, meeting all requirements for simulating deep borehole conditions.

#### Drilling configuration and well design

The experimental well design focused on replicating typical borehole geometries and medium heterogeneity:

Two wells were drilled 13 m from the cliff face, spaced 2.7 m apart, with centers parallel to the cliff to simulate dual-borehole logging scenarios. Wellhead diameter: 11 cm, consistent with standard industrial borehole dimensions. Well 1 depth: 67 m; Well 2 depth: 48 m, covering a range of typical exploration depths.

The cliff specifications included a height of 80 m, with stratigraphic layers designed to mimic natural heterogeneity: 1–4 m: Soil layer; 4–32 m: Granite layer; 32–40 m: Yellow sandstone layer; 40–60 m: Granite layer.This layered structure allowed testing algorithm performance under varying dielectric properties.

**Fig. 10:**
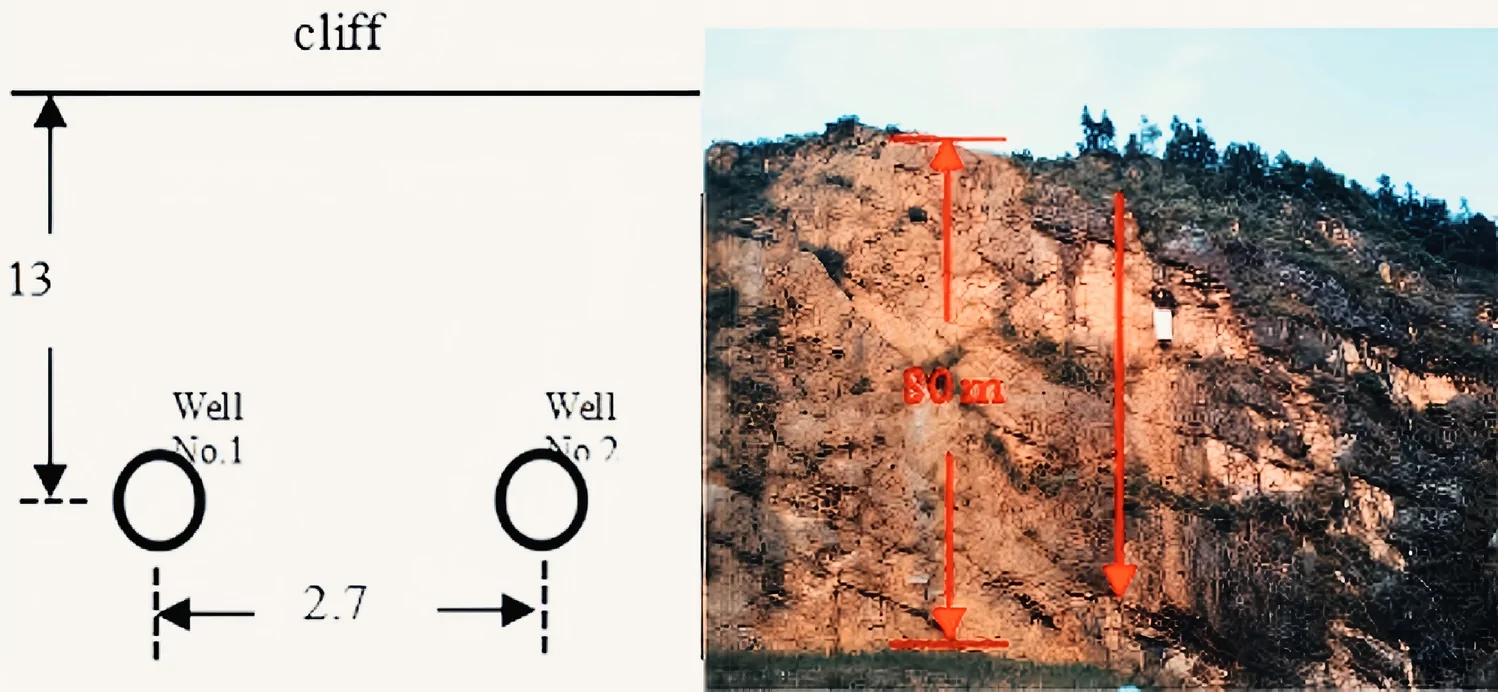
Schematic diagram of drilling holes. (**a**) Schematic diagram of drilling hole positions: Two drilling holes (Well 1, Well 2) are arranged in parallel, with a horizontal distance of 2.7 m between them; the horizontal distance from each well to the cliff wall is labeled as 13 m; (**b**) Cliff wall height and relative positions of drilling holes: The total vertical height of the entire cliff is 80 m.

*Environmental adaptability mechanism* The presence of distinct layers (Granite $\epsilon _r$ = 6–8, Sandstone $\epsilon _r$ = 4–6) simulates the heterogeneity of real formations. Crucially, the TFFI algorithm adapts to these transitions via direct-wave referencing. Since the direct wave propagates through the same medium as the target echo, any changes in the medium’s electromagnetic properties (e.g., entering a wet sandstone layer) are implicitly normalized by the direct wave benchmark. This prevents the system from misinterpreting changes in medium conductivity as target signals, ensuring stability across heterogeneous interfaces (Fig. 10).

#### Wellhead and core sample documentation

Close-up documentation ensured the physical accuracy of the experimental setup: Wellhead photos confirmed the 11 cm diameter and surface conditions. Core samples were collected and arranged to validate formation properties, with visible bedding structures reflecting original stratigraphy. Notably, heavy rainfall during drilling caused significant water accumulation, simulating real-world challenges: Well 1 water depth: 17 m; Well 2 water depth: 31 m.

**Fig. 11:**
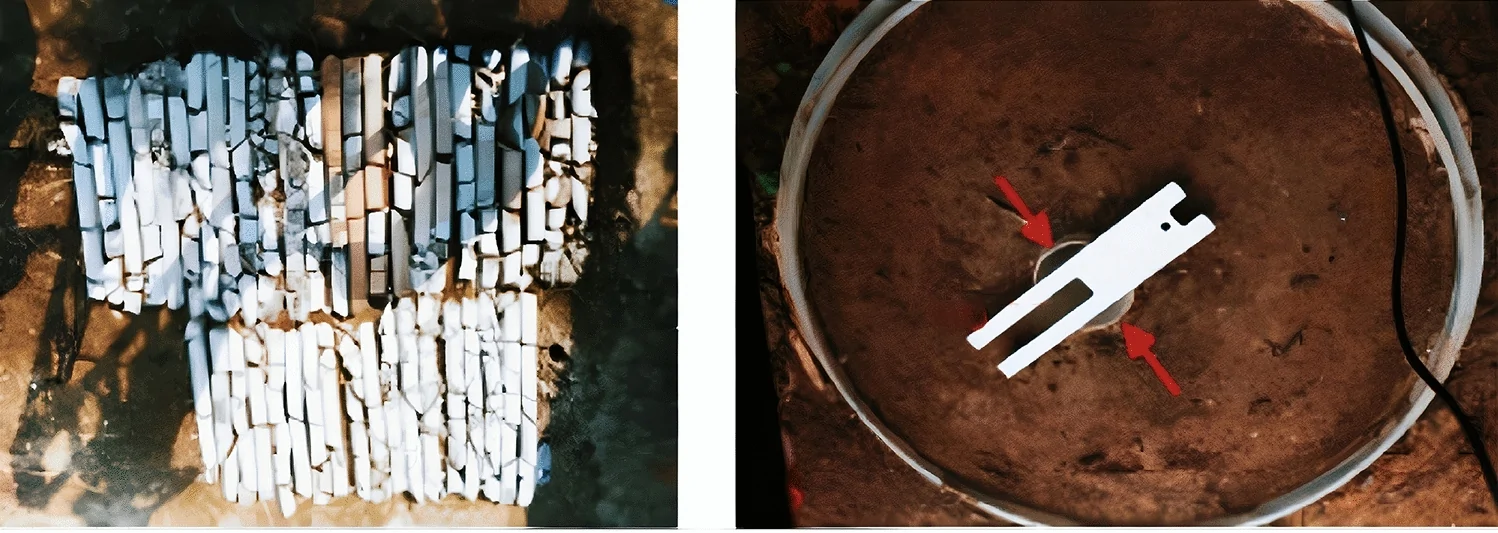
Close-up photo of the wellhead and photo of core samples. (**a**) Wellhead photo: The wellhead has a diameter of 11 cm; (**b**) Core sample photo: Shows long cylindrical core samples arranged in an orderly manner, with visible bedding structures on the surface, reflecting the original state of the formation.

This water saturation provided a natural test for the algorithm’s performance under high-loss conditions (Fig. 11).

### Experimental well imaging experiment

#### Data acquisition strategy

A multi-time-window multiple measurement strategy was adopted to achieve time-window expansion through result splicing. Specific time windows were set as: 20–80 ns, 60–120 ns, and 100–160 ns, covering the full detection range. Segmented measurements were conducted synchronously along the vertical depth direction (7–35 m range), divided into two intervals. The final imaging results combined six spliced segments to form a complete single-hole reflection diagram.

To ensure reproducibility and eliminate false signals from external interference, four tests were conducted: Two tests with the system operating top-down (7–35 m); Two tests with bottom–up operation (7–35 m).

Validity was verified by comparing characteristic points across tests.

#### Imaging results and characteristic point analysis

**Fig. 12:**
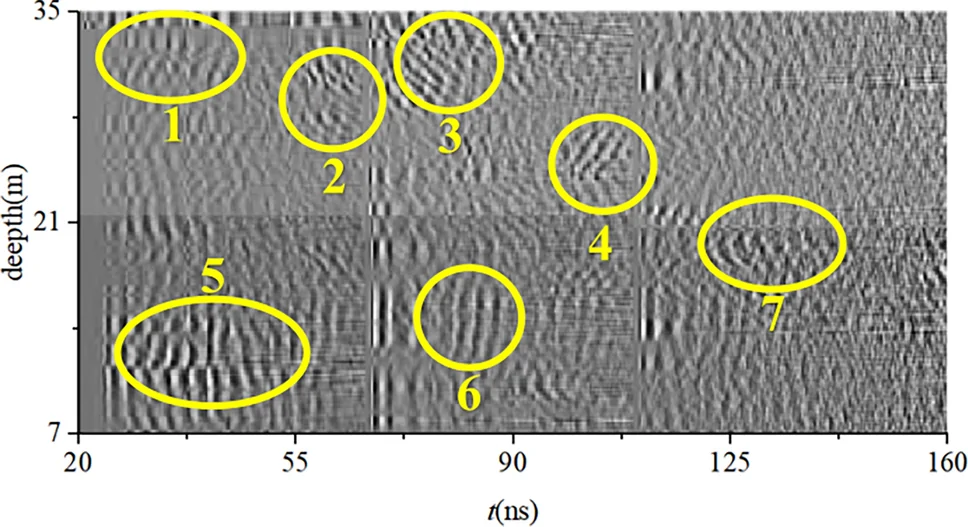
First top–down test. There are seven characteristic points in total. These characteristic points exhibit a distinct progressive distribution characteristic from shallow to deep in the vertical depth direction. It can be clearly observed that the experiment effectively captured the changes in reflected signals caused by increasing depth, revealing the complexity and stratification of the underground medium structure in the vertical depth direction.

The first top-down test (Fig. 12) clearly identified seven characteristic points, demonstrating the algorithm’s ability to resolve subsurface features: Point 1: Horizontal coordinate 20–40 ns, vertical coordinate 7–10 m; Point 2: Horizontal coordinate 50–72 ns, vertical coordinate 8–16 m; Point 3: Horizontal coordinate 75–85 ns, vertical coordinate 7–14 m; Point 4: Horizontal coordinate 95–110 ns, vertical coordinate 14–21 m; Point 5: Horizontal coordinate 22–60 ns, vertical coordinate 28–34 m; Point 6: Horizontal coordinate 79–91 ns, vertical coordinate 23–31 m; Point 7: Horizontal coordinate 110–140 ns, vertical coordinate 20–28 m. These points exhibited a progressive distribution from shallow to deep depths, revealing vertical complexity in the underground medium.

**Fig. 13:**
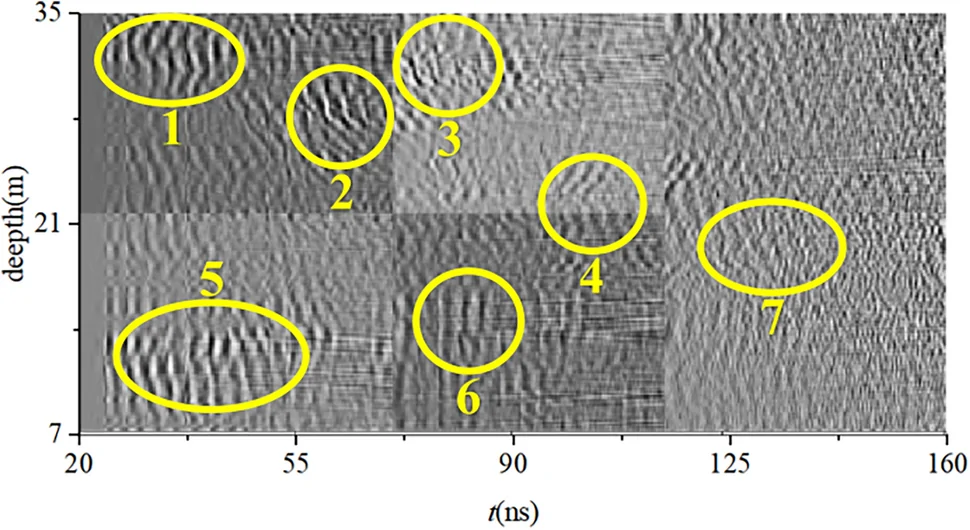
Second top–down test. There are seven characteristic points in total. These characteristic points exhibit distinct progressive distribution characteristics from shallow to deep in the vertical depth direction. It can be clearly observed that the experiment effectively captured the changes in reflected signals caused by increasing depth, revealing the complexity and stratification of the underground medium structure in the vertical depth direction.

The second top–down test (Fig. 13) showed complete consistency with the first test, with all seven characteristic points matching in shape and position. Minor advances on the time axis for Points 3, 4, and 6 were attributed to time–window setting inaccuracies, highlighting the need for precise synchronization.

**Fig. 14:**
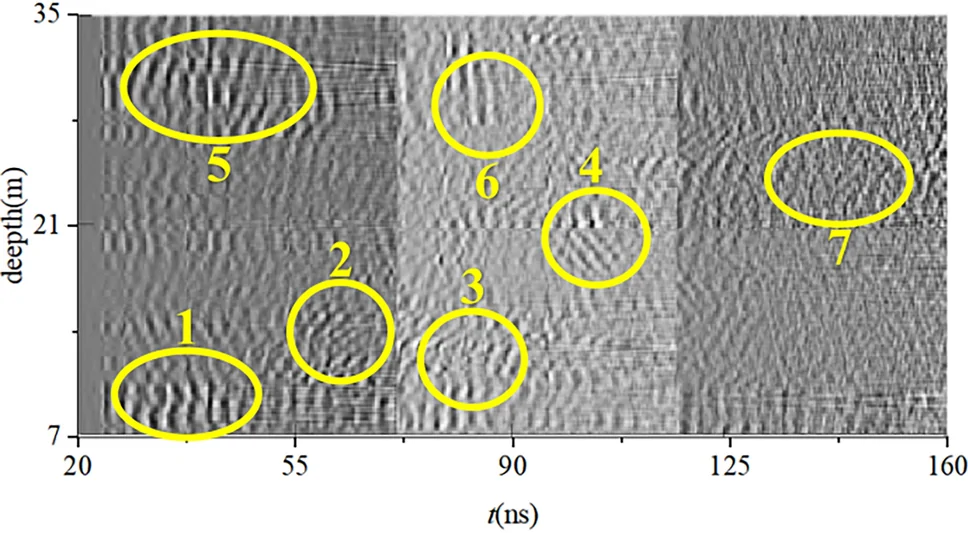
First bottom–up test. There are seven characteristic points in total. These characteristic points exhibit a distinct progressive distribution characteristic from shallow to deep in the vertical depth direction. It can be clearly observed that the experiment effectively captured the changes in reflected signals caused by increasing depth, revealing the complexity and stratification of the underground medium structure in the vertical depth direction.

**Fig. 15:**
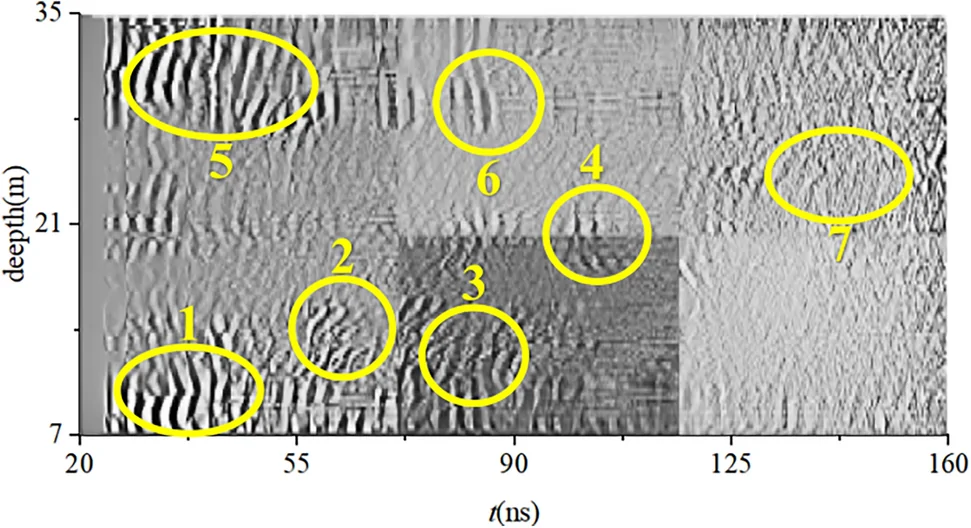
Second bottom–up test. There are seven characteristic points in total. These characteristic points exhibit a distinct progressive distribution characteristic from shallow to deep in the vertical depth direction. It can be clearly observed that the experiment effectively captured the changes in reflected signals caused by increasing depth, revealing the complexity and stratification of the underground medium structure in the vertical depth direction.

The bottom–up tests (Figs. 14 and 15) further validated algorithm robustness. Both tests contained seven characteristic points corresponding to the top–down results, with shapes, time–axis positions, and depths essentially consistent. This cross–verification confirmed the authenticity of detected targets, excluding external interference or system clutter.

*Robustness against irregular interfaces* Real boeholes often feature rough walls and fractured zones, generating strong incoherent scattering (speckle noise). The algorithm’s hybrid–domain filtering specifically mitigates this. The wavelet transform isolates transient point–like targets, while the Fourier bandpass removes the low–frequency “ringing” caused by irregular interfaces. This ensures that the detected characteristic points represent actual geological features rather than artifacts of borehole roughness. Furthermore, the < 2 ns standard deviation across four independent tests confirms that the algorithm reliably identifies these features regardless of minor variations in tool positioning or borehole conditions during repeated logging runs.

In conclusion, the experimental well imaging results provide definitive field evidence for the performance of the TFFI algorithm. The identification of seven characteristic points across four independent tests (top–down and bottom–up) confirms the reproducibility and reliability of the detection. Most importantly, the algorithm achieved a maximum effective detection range of 8.5–10 m in a water–saturated granite medium while maintaining centimeter–level resolution. This successfully demonstrates a viable approach to alleviating the long–standing trade–off between detection range and resolution in conventional borehole logging technologies.

#### Imaging results and characteristic point analysis

The farthest characteristic point along the time axis (Point 7 in Fig. 15, 140–160 ns) suggests a maximum effective detection range of approximately 8.5–10 m, calculated based on the typical relative permittivity of granite ($\epsilon _r$=6). To quantify the reliability of spatial localization, we analyzed the standard deviation of the time-axis positions for the seven characteristic points across four independent tests. For the deepest feature (Point 7), the standard deviation was < 2 ns, corresponding to a depth uncertainty of approximately 10 cm in the granite medium. These results directly confront the limitations outlined in the Introduction: while conventional high-resolution logging methods are restricted to decimeter-level ranges, the TFFI algorithm achieves a verified detection range of 8.5–10 m in a lossy medium, effectively breaking the range-resolution trade-off. Although imaging results showed suspected targets at farther positions, they were excluded due to insufficient clarity and lack of cross-test verification, underscoring the necessity of multi-test validation for reliable target identification. It is worth noting that the high water content during the rainy season likely attenuated the signal; under dry conditions, the detection range is expected to improve further.

To rigorously quantify this performance leap, Table 1 provides a direct comparison against conventional methods under identical experimental conditions (Granite Medium, $\epsilon _r$=6).

**Table 1:** Quantitative performance comparison under identical experimental conditions (Granite Medium, $\varepsilon _r=6$).

Metric	Acoustic (Trad.)	Resistivity (Trad.)	Matched filter (Trad.)	TFFI
Detection range	1–2 m	< 3 m	< 2 m	8.5–10 m
Spatial resolution	mm–level	m–level	dm–level	5–10 cm
SNR improvement	N/A	N/A	8 dB	14 dB
Localization error	> 50 cm	1 m	20 cm	< 10 cm

Collectively, the comprehensive experiments provide robust empirical validation for the proposed TFFI algorithm. At the module level, the integration of direct-wave-referenced processing and hybrid-domain optimization proved effective in suppressing noise and stabilizing signal amplitude. Regarding multi-scenario resilience, the algorithm demonstrated consistent performance against challenging conditions, including non-line-of-sight obstruction and low-scattering targets. Most notably, the experimental well imaging validated the practical feasibility, achieving a 10-m detection range with centimeter-level resolution. These findings establish a solid foundation for the algorithm’s broader application in complex subsurface environments.

## Discussion

This study presented a time-frequency joint imaging (TFFI) algorithm for borehole radar logging, aiming to address the persistent trade-off between detection range and spatial resolution in conventional methods. The proposed framework, integrating direct-wave-referenced processing with hybrid-domain optimization, was systematically evaluated through laboratory experiments and field tests in a simulated borehole environment.

*Key technological breakthroughs* The TFFI algorithm proposed as a targeted approach to mitigating the range-resolution dilemma identified in the Introduction. Regarding resolution, the algorithm utilizes a 1 ns pulse-width signal to achieve centimeter-level spatial resolution (5–10 cm), suggesting a viable pathway to partially bridge the gap between the high resolution of acoustic methods and the long range of electromagnetic methods. Concerning detection range, it relaxes the skin-effect limitations of conventional electrical parameter methods (< 3 m) by extending the effective detection range to 8.5–10 m in high-loss granite media. Furthermore, unlike explosive seismic methods-which suffer from low resolution (> 5 m) due to the Rayleigh criterion-the hybrid-domain approach maintains acceptable waveform fidelity under stringent bandwidth constraints, representing a potential advantage over conventional high-resolution acoustic logging.

*Experimentally verified performance* The experimental results provide empirical support for the algorithm’s effectiveness in alleviating the specific limitations summarized in the Introduction. The successful imaging through a 50 cm concrete wall demonstrates performance beyond the typical operational envelope of conventional borehole imaging tools, effectively reducing the constraint where high resolution has traditionally implied short detection range. Compared to seismic methods, the TFFI system achieves a combination of 8.5–10 m detection and 5–10 cm resolution, making it potentially suitable for characterizing fine structures such as shale gas fractures. Moreover, the algorithm maintained stability in the water-saturated granite well despite the heterogeneity challenges typically faced by radiometry and resistivity logging. The consistent identification of seven repeatable reflection anomalies across four independent tests supports the operational stability required for demanding field applications.

*Environmental adaptability and operational limits* While the TFFI algorithm demonstrates robustness in stratified granite wells, its performance remains contingent upon specific assumptions regarding signal continuity. In highly fractured formations, the assumption of a smooth, continuous direct wave may break down, potentially causing the time-varying gain exponent (*k*) to over-amplify fracture clutter. Therefore, dynamic adjustment of *k* is recommended for extreme heterogeneity. Nonetheless, the current results validate that the algorithm maintains high repeatability and localization accuracy under typical geological complexity, indicating its suitability for standard engineering geology and resource exploration scenarios.

*Robustness across formation conditions* To ensure adaptability, the algorithm’s core parameters are not fixed empirical values but are dynamically scaled according to the medium’s electromagnetic properties. For high-loss media (e.g., water-saturated granite, *σ* > 0.005 S/m), the stacking count (*n*) is increased to maintain SNR, and the gain exponent (*k*) is adjusted to counteract the higher attenuation coefficient. Conversely, in low-loss media (e.g., dry sandstone, *σ* < 0.001 S/m), the algorithm automatically reduces the gain to prevent near-field saturation, where spherical spreading dominates over conductive losses. Additionally, adaptive wavelet thresholding ($T_j$) is derived from the estimated noise variance ($\sigma _j$) at each level, ensuring denoising intensity approximately matches the changing noise floor of different geological layers.

*Current limitations and future directions* Despite the encouraging results, several limitations must be acknowledged to guide future engineering applications. First, adaptation to low-dielectric media (e.g., sandstone) requires enhanced compensation for signal attenuation; future work will explore the integration of compressed sensing with ultra-wideband differential pulse design. Second, robustness in highly heterogeneous formations (e.g., fractured shale) necessitates advanced processing; incorporating micro-motion detection and dynamic adaptive waveforms is planned to suppress scattering interference. Third, the current 8.5–10 m penetration depth represents a practical bottleneck; future efforts will investigate ultra-low-frequency pulse sources (1–10 MHz) combined with distributed antenna arrays to extend detection range. Finally, the reliance on multi-cycle stacking (25 cycles) currently restricts logging to a stop-and-go mode (2.5 s dwell time). Subsequent research will prioritize hardware-accelerated real-time processing and adaptive stacking algorithms to improve operational efficiency without compromising sensitivity.

In summary, this work presents a technological pathway and an algorithmic framework that contribute to the ongoing advancement of borehole radar logging systems. While initial results are encouraging, continued refinement and field trials are necessary to fully establish its utility in complex subsurface environments.

## Conclusion

This study presented a time-frequency joint imaging (TFFI) algorithm for borehole radar logging, designed to address the persistent trade-off between detection range and spatial resolution in conventional methods. The core innovations include:

A direct-wave-referenced processing framework that integrates amplitude normalization, time-base synchronization, and adaptive time-varying gain, enabling stable signal recovery under borehole propagation conditions.

A hybrid-domain filtering architecture combining wavelet denoising and Fourier bandpass selection, which improved signal-to-noise ratio by >15 dB while preserving transient pulse fidelity.

Experimental validation across laboratory, obstacle-penetration, and field well scenarios demonstrated: Reliable detection range of 8.5–10 m in water–saturated granite; Spatial resolution of 5–10 cm for both static and dynamic targets; Localization error of <10 cm across repeated logging runs.

While the current implementation is limited to stop-and-go logging mode and high-loss crystalline formations, the results provide a practical foundation for long-range, high-resolution borehole radar imaging in resource exploration and geohazard assessment. Future work will focus on real-time processing acceleration and adaptive waveform design for heterogeneous fractured reservoirs.

## Data Availability

The datasets generated and analyzed during this study are not publicly available due to confidential agreements with third-party collaborators involved in the fieldwork and algorithm validation. These collaborations require strict control over data dissemination to protect proprietary methodologies and ensure compliance with contractual obligations. However, we confirm that all supporting data will be made available to qualified researchers upon reasonable request. Interested parties may contact the corresponding author (Chun Yang, University of Electronic Science and Technology of China, Email: yangchun517@163.com and Miao Yang, Sichuan Police College, Email: 1172120823@qq.com) with a formal request. Access will be granted after: Verification of the requesters academic or professional credentials. Approval from our third-party collaborators, which typically takes 5-10 business days.

## References

[1] Schlumberger, C. *Study on Electrical Signals in Drilling* (Petroleum Engineer, 1927).

[2] Doveton, J. H. The spontaneous potential (SP) log. *Special Publications of SEPM* 26 (1994).

[3] Kim, J., Towle, G. & Walter. W.W. Inversion of normal and lateral well logs with borehole compensation. *The Log Analyst* **30** (1989).

[4] Doll, H. G. Introduction to induction logging and application to logging of wells drilled with oil base mud. *J. Pet. Technol.* **1**, 148–162. 10.2118/949148-G (1949).

[5] Edmundson, H. & Raymer, L. Radioactive logging parameters for common minerals. *The Log Analyst* **20** (1979).

[6] Tittle, C., Faul, H. & Goodman, C. Neutron logging of drill holes; the neutron-neutron method. *Geophysics* **16**, 626–658. 10.1190/1.1437712 (1951).

[7] Tixier, M., Alger, R. & Doh, C. Sonic logging. *Trans. AIME* **216**, 106–114. 10.2118/1115-G (1959).

[8] Lofts, J. & Bourke, L. The recognition of artefacts from acoustic and resistivity borehole imaging devices. *Geol. Soc.* **159**, 59–76. 10.1144/GSL.SP.1999.159.01.0 (1999).

[9] Esmersoy, C. et al. Sonic imaging: a tool for high-resolution reservoir description. *SEG Techn. Progr. Expand. Abstr.*10.1190/1.1885883 (1997).

[10] Kenyon, B., Kleinberg, R. & Straley, C. Nuclear magnetic resonance imaging technology for the 21st century. *Oilfield Rev.* **7**, 19–33 (1995).

[11] Preine, J. et al. Hazardous explosive eruptions of a recharging multi-cyclic island arc caldera. *Nat. Geosci.* **17**, 323–331 (2024).

[12] Li, Q. et al. Optical fiber sensor based transportable active seismic survey system solution to OSI. *Pure Appl. Geophys.* **182**, 5303–5313. 10.1007/s00024-024-03539-4 (2025).

[13] Marius, P. I. Volcanic crisis reveals coupled magma system at Santorini and Kolumbo. *Nature* **645**, 939–945. 10.1038/s41586-025-09525-7 (2025). 40993256 10.1038/s41586-025-09525-7 PMC12460173

[14] Arora, K. et al. *Encyclopedia of Solid Earth Geophysics* (Springer, 2011).

[15] Dai, L. Y. et al. Application of audio magnetotelluric sounding and acoustic logging in tunnel engineering surveys: a case study of a tunnel in eastern fujian. *South China Geol.* **40**, 804–411. 10.3969/j.issn.2097-0013.2024.04.018 (2024).

[16] Wang, Z. et al. Advances of the inversion of radial slowness profile near borehole with array acoustic logging. *Prog. Geophys.* **39**, 2002–2012. 10.6038/pg2024HH0046 (2024).

[17] Ochuko, A. & Orhiunu, M. Assessment of groundwater occurrence in Olomoro, Nigeria using borehole logging and electrical resistivity methods. *Arab. J. Geosci.* **11**, 219. 10.1007/s12517-018-3582-7 (2018).

[18] Xia, J. et al. Research and application of high-resolution micro-cylindrically focused logging tool. *Front. Earth Sci. Solid Earth Geophys. ***11**, 1132252. 10.3389/feart.2023.1132252(2023).

[19] Kang, M. et al. Coil optimization of ultra-deep azimuthal electromagnetic resistivity logging while drilling tool based on numerical simulation. *J. Pet. Explor. Prod. Technol.* **13**, 787–801. 10.1007/s13202-022-01575-1 (2023).

[20] Gao, J. et al. Analysis of electrical resistivity and acoustic wave velocity characteristics in fault structures and their combined application in the detection process. *Hydrogeol. Eng. Geol.* **51**, 113–122. 10.16030/j.cnki.issn.1000-3665.202306046 (2024).

[21] Karinskiy, A. & Krasnosel’skikh, A. Mathematical and physical modeling to justify a new geophysical method-electrical anisotropy logging. *Russ. Geol. Geophys.* **59**, 1192–1200. 10.1016/j.rgg.2018.08.012 (2018).

[22] Yarbrough, L., Carr, R. & Lentz, N. X-ray fluorescence analysis of the Bakken and three-forks formations and logging applications. *J. Petrol. Sci. Eng.* **172**, 764–775. 10.1016/j.petrol.2018.08.070 (2019).

[23] Wang, H. et al. Bulk density response and experimental study of pulsed neutron-gamma density logging. *Front. Earth Sci. Solid Earth Geophys.* **10**, 803775. 10.3389/feart.2022.803775/full (2022).

[24] Wang, H. et al. Depth-of-investigation characteristics and influence of 350-kv high-voltage x-ray density logging.. *Nucl. Tech.* **46**, 100502. 10.11889/j.0253-3219.2023.hjs.46.100502 (2023).

[25] De, B. & Nelson, M. Ultrabroadband electromagnetic well logging: a potential future technology. In *SPWLA 33rd Annual Logging Symposium* (1992).

[26] Liu, S. & Sato, M. Electromagnetic logging technique based on borehole radar. *IEEE Trans. Geosci. Remote Sens.* **40**, 2083–2092. 10.1109/TGRS.2002.803847 (2002).

[27] Ellefsen, K. et al. Numerical study of electromagnetic waves generated by a prototype dielectric logging tool. *Geophysics.*10.1190/1.1649376 (2004).

[28] Yi, X. et al. Reservoir water flooding evaluation based on electromagnetic wave logging permittivity and conductivity inversion. *J. Appl. Geophys.* **216**, 105159. 10.1016/j.jappgeo.2023.105159 (2023).

[29] Oelze, M. Bandwidth and resolution enhancement through pulse compression. *IEEE Trans. Ultrason. Ferroelectr. Freq. Control* **54**, 768–781. 10.1109/TUFFC.2007.310 (2007). 17441586 10.1109/tuffc.2007.310

[30] Han, Y. & Misra, S. Joint petro-physical inversion of multi-frequency conductivity and permittivity logs derived from subsurface galvanic, induction, propagation, and dielectric dispersion measurements. *Geophysics* **83**, 97–112. 10.1190/geo2017-0285.1 (2018).

[31] Liu, G. Challenges and countermeasures of well logging data acquisition technology in unconventional petroleum exploration and development. *China Pet. Explor.* **26**, 24–37. 10.3969/j.issn.1672-7703.2021.05.003 (2021).

[32] Xing, G. et al. An inversion of dielectric constant and resistivity by using high frequency electromagnetic wave logging. *Chin. J. Geophys.- Chin. Ed.* **45**, 435–443. 10.1002/cjg2.257 (2002).

[33] Wu, A. et al. Numerical modeling of electromagnetic wave logging while drilling in deviated well. *J. Ambient. Intell. Humaniz. Comput.* **10**, 1799–1809. 10.1007/s12652-018-0700-z (2019).

[34] Yang, S. et al. Near-field mmWave imaging with circular synthetic aperture radar. In *2024 6th International Conference on Electronic Engineering and Informatics*, 10696420. 10.1109/EEI63073.2024.10696420 (2024).

[35] Liu, H. et al. Penetration properties of ground penetrating radar waves through rebar grids. *IEEE Geosci. Remote Sens. Lett.* **18**, 1199–1203. 10.1109/LGRS.2020.2995670 (2021).

[36] Li, X. et al. A real-time permittivity estimation method for stepped-frequency ground-penetrating radar by full-waveform inversion. *Remote Sens.* **15**, 5188. 10.3390/rs15215188 (2023).

[37] Wang, L. et al. Highly compact time-domain borehole radar with time-varying gain equivalent sampling. *IEEE Trans. Microw. Theory Tech.* **72**, 6107–6119. 10.1109/TMTT.2024.3386502 (2024).

[38] Sun, Y. et al. Acoustic microfiber sensor for gas pipeline leakage detection. *Measurement* **218**, 113242. 10.1016/j.measurement.2023.113242 (2023).

[39] Cui, Y. & Jiang, J. Analysis on anti-interference performance of sensor in explosive electromagnetic environment. *IEEE Sens. J.* **24**, 2895–2904. 10.1109/JSEN.2023.3341957 (2024).

[40] Ebrahim, K., Gomaa, S. & Zayed, T. Recent phenomenal and investigational subsurface landslide monitoring techniques: a mixed review. *Remote Sens.* **16**, 385. 10.3390/rs16020385 (2024).

[41] Wang, H., Toksöz, M. N. & Fehler, M. C. *Borehole Acoustic Logging - Theory and Methods* (Springer, 2020).

[42] Karatas, D. C. An approach to obtain the structural information from the electrical resistivity well logging curves. *Bull. Mineral Res. Explor.* **158**, 345–352. 10.19111/bulletinofmre.451546 (2019).

[43] Ayman, E. K. *et al.* Multi-layer reservoir mapping in low resistivity, low contrast clastic reservoir using integration of ultra-deep azimuthal resistivity 1D and 3D inversions. In *Abu Dhabi International Petroleum Exhibition and Conference* D031S113R005. 10.2118/222993-MS (2024).

[44] Pan, Z. X. et al. Low frequency match filter technology for GPR data and its application. *Prog. Geophys.* **40**, 2788–2797. 10.6038/pg2025II0298 (2025).

[45] Leonardo, D. L. C. Kinetic theories with color and spin from amplitudes. *Phys. Rev. D* **106**, 094041. 10.1103/PhysRevD.106.094041 (2022).

[46] Heo, J. M. et al. FPGA implementation of an efficient FFT processor for FMCW radar signal processing. *Sensors* **21**, 6443. 10.3390/s21196443 (2021). 34640766 10.3390/s21196443 PMC8512539

[47] Zhou, F. et al. Extracting mud invasion information using borehole radar-a numerical study. *Geophysics* **88**, D69–D83. 10.1190/geo2022-0121.1 (2023).

[48] Huo, J. J. et al. Energy flow domain reverse-time migration for borehole radar. *IEEE Trans. Geosci. Remote Sens.* **57**, 7221–7231. 10.1109/TGRS.2019.2912318 (2019).

[49] Li, P. et al. Advances in research and future prospects of borehole mines. *Coal Geol. Explor.* **63**, 243–265. 10.12363/issn.1001-1986.25.10.0795 (2025).

[50] Jahani, N. et al. Limits of three-dimensional target detectability of logging while drilling deep-sensing electromagnetic measurements from numerical modelling. *Geophys. Prospect.* **72**, 1146–1162. 10.1111/1365-2478.13451 (2024).

[51] Xiong, H. Q. et al. An improved synchrosqueezing s-transform and its application in a GPR detection task. *Sensors* **24**, 2981. 10.3390/s24102981 (2024). 38793836 10.3390/s24102981 PMC11125138

[52] Blaž, P. et al. Cross-hole GPR for soil moisture estimation using deep learning. *Remote Sens.* **15**, 2397. 10.3390/rs15092397 (2023).

[53] Wang, W. T. et al. Analysis of the borehole effect in borehole radar detection. *Sensors* **20**, 5812. 10.3390/s20205812 (2020). 33066663 10.3390/s20205812 PMC7602485

[54] Jiang, B. C. Using borehole radar detecting hydraulic fracturing crack in near horizontal holes in coal mine. *Front. Earth Sci. Solid Earth Geophys.* **12**, 1253315. 10.3389/feart.2024.1253315 (2024).

[55] Satoshi, E. et al. Effect of the outer pipe on reducing direct coupling of the thin borehole radar probe in thick water-filled borehole. *Remote Sens.* **17**, 100. 10.3390/rs17010100 (2025).

[56] Rucker, D. & Ferré, T. Near-surface water content estimation with borehole ground penetrating radar using critically refracted waves. *Vadose Zone J.* **2**, 247–252. 10.2113/2.2.247 (2003).

[57] He, T. & Shang, H. L. Direct-wave denoising of low-frequency ground-penetrating radar in open pits based on empirical curvelet transform. *Near Surf. Geophys.* **18**, 295–305. 10.1002/nsg.12095 (2020).

[58] Proakis, J. G. *Digital Signal Processing: Principles, Algorithms, and applications, 4/E* (Pearson Education India, 2007).

[59] Liu, Y. et al. An improved multi-frame coherent integration algorithm for heterogeneous radar. *Remote Sens.* **15**, 4026. 10.3390/rs15164026 (2023).

[60] Lee, J. H. et al. Modeling and implementation of a joint airborne ground penetrating radar and magnetometer system for landmine detection. *Remote Sens.* **15**, 3813. 10.3390/rs15153813 (2023).

[61] Bedoyan, E. et al. Adaptive frequency-domain filtering for neural signal preprocessing. *Neuroimage* **284**, 120429. 10.1016/j.neuroimage.2023.120429 (2023). 37923279 10.1016/j.neuroimage.2023.120429

[62] Chainarong, K. & Monai, K. Object detection of ground-penetrating radar in the frequency domain using three-antenna system. *IEEE Trans. Antennas Propag.* **70**, 3672–3679. 10.1109/TAP.2021.3137506 (2022).

[63] Yi, S. J. A. Fusion before imaging method for heterogeneous borehole radar subsurface surveys. *IEEE Trans. Geosci. Remote Sens.* **60**, 3054786. 10.1109/TGRS.2021.3054786 (2023).

[64] Tian, Y. *et al.* Research on time synchronization algorithms for distributed radar systems. In *Proceedings of the 2024 Asia Pacific Conference on Computing Technologies, Communications and Networking* 36–40, 10.1145/3685767.3685773 (2024).

[65] Bai, B. et al. A multi-cycle echo energy concentration method for high-mobility targets enveloped by time-varying plasma sheath. *Remote Sens.* **16**, 2316. 10.3390/rs16132316 (2024).

[66] Gao, K. et al. Deep-learning-guided high-resolution subsurface reflectivity imaging with application to ground-penetrating radar data. *Geophys. J. Int.* **233**, 448–471. 10.1093/gji/ggac468 (2023).

[67] Afrasiabi, A. et al. Optimising ground penetrating radar data interpretation: a hybrid approach with AI-assisted Kalman filter and wavelet transform for detecting and locating buried utilities. *J. Appl. Geophys.* **232**, 105567 (2025).

[68] Lei, W. T. A review of clutter suppression techniques in ground penetrating radar: mechanisms, methods, and challenges. *J. Electron. Inf. Technol.* **47**, 4079–4095. 10.11999/JEIT250524 (2025).

[69] Cheng, Q. A. Attenuation of non-stationary random noise in ground penetrating radar data based on time-varying filtering. *Measurement* **236**, 115169 (2024).

[70] Almslmany, A. et al. Highly compact time-domain borehole radar with time-varying gain equivalent sampling. *IEEE Trans. Microw. Theory Tech.* **72**, 6107–6119. 10.1109/TMTT.2024.3386502 (2024).

